# Different Aggregation Pathways and Structures for Aβ40 and Aβ42 Peptides

**DOI:** 10.3390/biom11020198

**Published:** 2021-01-30

**Authors:** Li Wang, Kilho Eom, Taeyun Kwon

**Affiliations:** 1 Biomechanics Laboratory, College of Sport Science, Sungkyunkwan University (SKKU), Suwon 16419, Korea; wlchem621@gmail.com; 2 SKKU Advanced Institute of Nano Technology (SAINT), Sungkyunkwan University (SKKU), Suwon 16419, Korea

**Keywords:** Alzheimer’s disease, amyloid beta (Aβ), spherical oligomers, aggregation mechanism, morphology

## Abstract

Self-aggregation of amyloid-β (Aβ) peptides has been known to play a vital role in the onset stage of neurodegenerative diseases, indicating the necessity of understanding the aggregation process of Aβ peptides. Despite previous studies on the aggregation process of Aβ peptides, the aggregation pathways of Aβ isoforms (i.e., Aβ40 and Aβ42) and their related structures have not been fully understood yet. Here, we study the aggregation pathways of Aβ40 and Aβ42, and the structures of Aβ40 and Aβ42 aggregates during the process, based on fluorescence and atomic force microscopy (AFM) experiments. It is shown that in the beginning of aggregation process for both Aβ40 and Aβ42, a number of particles (i.e., spherical oligomers) are formed. These particles are subsequently self-assembled together, resulting in the formation of different shapes of amyloid fibrils. Our finding suggests that the different aggregation pathways of Aβ isoforms lead to the amyloid fibrils with contrasting structure.

## 1. Introduction

Neurodegenerative diseases such as Alzheimer’s, Parkinson’s, and Huntington’s disease have recently received much attention in our society because of their devasting effect on human cognitive ability or memory [1]. They are attributed to the deposition of amyloidogenic proteins, which increases the toxicity to nervous system [2,3]. These deposited amyloidogenic proteins exist in the form of a macromolecular structure, referred to as amyloid structure, that is locally deposited in organs or tissues because of the aggregation of denatured or misfolded proteins [4,5], and this amyloid structure is a very stable substance and not easily degraded once formed [4,6]. The amyloid structure made from aggregation of amyloidogenic proteins can exist in the different sized structural forms ranging from oligomer to protofibril and fibril, while the oligomers with their size of ~10 nm have recently been reported to be cytotoxic to functional cells [7,8].

Among neurodegenerative diseases, Alzheimer’s disease (AD) is the major cause of dementia, and has recently been reported to gradually appear in younger people leading to great social repercussions [9,10]. The AD is originated from the aggregation of β amyloid (abbreviated as Aβ) proteins resulting in the formation of amyloid structures. This Aβ protein present in the brain is categorized into either of two types such as Aβ40 and Aβ42, while the majority is Aβ40 [11]. Despite only two sequence differences between Aβ40 and Aβ42, which are isoleucine and alanine in the C-terminus, Aβ42 has been found to be responsible for the onset of AD [12]. In particular, Aβ42 aggregates exhibit higher toxicity than Aβ40 aggregates because of the faster growth of Aβ42 aggregates. In recent years, the different aggregation kinetic mechanisms of Aβ40 and Aβ42 have been reported [13,14,15,16]. However, those results are limited in understanding the detailed mechanism of aggregation process to form the amyloid structures. Specifically, the structural characteristics of Aβ aggregates formed by two types of Aβ during the aggregation process have not been identified. In addition, as the self-aggregation process determines the structures of amyloid aggregates and their physical properties [17,18,19], it is necessary to study how the aggregation process is related to the structural characteristics of amyloid aggregates.

In this work, we study the detailed mechanism of different aggregation pathways for two types of Aβ and their related structures. Specifically, we visualized the aggregation process of Aβ40 and Aβ42 by using atomic force microscopy (AFM), which is able to image the small-scale proteins and protein assemblies such as oligomers and fibrils. Our result may shed light on the underlying mechanism of aggregation pathways for Aβ, which provides further insight into how to regulate the Aβ aggregation for the therapeutics of neurodegenerative diseases.

## 2. Methods and Materials

### 2.1. Materials

Aβ42 (MDAEFRHDSGYEVHHQKLVFFAEDVGSNKGAIIGLMVGGVVIA), and Aβ40 (MDAEFRHDSGYEVHHQKLVFFAEDVGSNKGAIIGLMVGGVV) peptides with the purity higher than 95% were purchased from AnaSpec, Inc. (San Jose, CA, USA) The 1,1,1,3,3,3-hexafluoro-2-isopropanol (HFIP) was purchased from Sigma–Aldrich (St. Louis, MO, USA). All the other chemical agents used in this paper were obtained from Sigma–Aldrich. The peptides and chemical agents were used as purchased without further treatment.

### 2.2. Solution Preparation

Aβ42 and Aβ40 were prepared as previously described. Briefly, Synthetic Aβ42 and Aβ40 were dissolved in HFIP solution at a concentration of 1 mg/mL. The solution was sonicated in water bath for 5 min to break up any pre-existing aggregates and then aliquoted into 20 µL portions. The HFIP was removed in the vacuum desiccator overnight and the samples were stored at −20 °C before use. A 25 mM phosphate buffer at pH 7.4 with 25 mM NaCl was prepared and used for all experiments.

### 2.3. Thioflavin-T Fluorescence Assay

To investigate the aggregation behaviors of amyloid, the Thioflavin-T (ThT) fluorescence assays were performed on a microplate reader equipped with a 440 nm excitation filter, and a 482 nm emission filter at 37 °C. The ThT fluorescence was recorded at a step of 5 mins after shaking plates for 5 s. The sodium phosphate buffer used for preparing the experimental samples was supplemented with 5 mM ThT from a concentrated stock. All solutions were kept inside ice before measurements. Each experiment was repeated at least twice in different plates.

### 2.4. Atomic Force Microscopy Imaging

Microstructures were characterized using a high-resolution atomic force microscopy (BioScope Catalyst, Bruker Inc., Fremont, CA, USA). In a typical process, 10 μL of a sample containing Aβ was deposited onto freshly cleaved surface of muscovite mica, and was kept stable for 5 min. The mica was then washed with 50 μL deionized water and dried under dynamic vacuum overnight. Following deposition, the mica substrate was ubiquitously covered by samples. AFM images were acquired under both tapping mode and scanAsyst mode at scanning rate of 0.6 Hz. The acquired images were used to examine the diameter distribution and to calculate the persistence length of the fibrils. For analysis, we used a Nanoscope software (Ver. 6.14, VEECO, San Jose, CA, USA) to measure the diameter of fibrils from their height profiles acquired from corresponding AFM images. The contour length (*L*) of the fibrils and the end-to-end distance (*R*) between the fibril ends were measured with the Simple Neurite Tracer in Fiji (*n* ≥ 100) [20]. The persistence length, *P*, is calculated according to the relation of <*R*2>_2D_ = 4*PL*[1 -2*P*/*L*(1–e–*L*/2*P*)] [21]. To measure the values with greater statistical accuracy, we averaged more than 100 fibrils for each sample.

## 3. Results and Discussion

**Figure 1:**
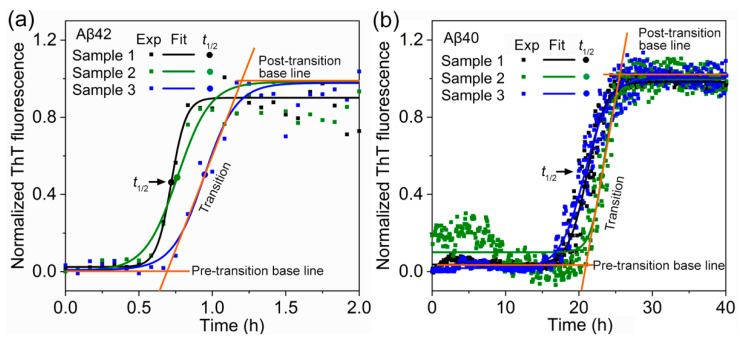
ThT-fluorescence spectrum as a function of incubation time for a solution containing (**a**) Aβ42 or (**b**) Aβ40, at a concentration of 10 μM for each solution.

To understand the aggregation kinetics of Aβ40 and Aβ42 monomer, we performed time-resolved thioflavin T (ThT) fluorescence experiments. Figure 1 displays the ThT fluorescence signal as a function of incubation time for the Aβ42 and Aβ40 monomeric solutions (with concentration of 10 μM). The ThT spectra of Aβ42 and Aβ40 are well fitted to a commonly used empirical sigmoidal function given by *y*(*t*) = *y*_0_ + *A*/[1 + exp{–*k*(*t* – *t*_0.5_)}], where *y*(*t*) is a time-dependent ThT signal, and *t*_0.5_ is the half-time defined as the time in which the ThT signal relative to pre-transition baseline reaches 50% of the amplitude of transition. Here, it should be noted that the ThT signal does not directly reflect the microscopic kinetic model of amyloid fibrillation, because ThT fluorescence experiment is based on the bulk measurement of overall fibrils over time at macroscopic level [22]. Specifically, even in the pre-transition baseline of ThT fluorescence (see Figure 1), there may exist the amyloid nuclei and/or fibrils, but their amount is not sufficient to be detected by ThT molecules.

For a quantitative comparison between Aβ40 and Aβ42 aggregation, we measured the half-time of these aggregations, since the half-time is determined by the overall aggregation rate. The half-time of Aβ42 ThT signal is measured as 0.81 ± 0.12 h, which is markedly smaller than the half-time of Aβ40 ThT signal (i.e., 21.75 ± 1.36 h). In fact, it should be noted that the fibrillation process of Aβ40 is significantly slowed down in comparison with the previous result [14], reporting that it takes less than 5 h for Aβ40 monomers with a concentration of 9 μM to be fully converted into fibrils in a 1 mL solution containing 6 M GuHCl. The slow fibrillation in this work is because we intentionally choose a solution that is able to reduce the reaction rate, which allows us to study the structure of Aβ aggregates at each stage using AFM.

**Figure 2:**
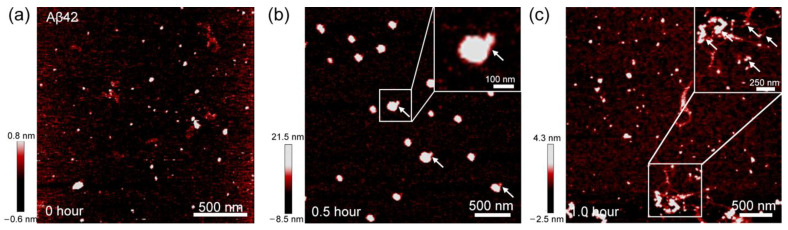
AFM images for Aβ42 aggregates that are formed under the incubation time of (**a**) 0 h, (**b**) 0.5 h, and (**c**) 1 h, respectively. The white arrows indicate the small spherical particle attached into large one.

It should be noted that ThT spectra only provide the limited information about the fibrillation process. Specifically, though ThT assay provides the kinetics of aggregation at macroscopic level, it does not enable an insight into the microstructure evolution during aggregation process. Alternatively, we unveiled the mechanism of microstructure evolution during the fibrillation process by employing AFM that is able to directly visualize the morphology of Aβ aggregates at each fibrillation phase. Figure 2a–c shows the AFM images of the Aβ42 aggregates obtained based on incubation of Aβ42 monomers in phosphate buffer for an incubation time of 0, 0.5, and 1 h, respectively, corresponding to the pre-transition baseline, transition, and post-transition baseline of ThT signal, respectively.

**Figure 3:**
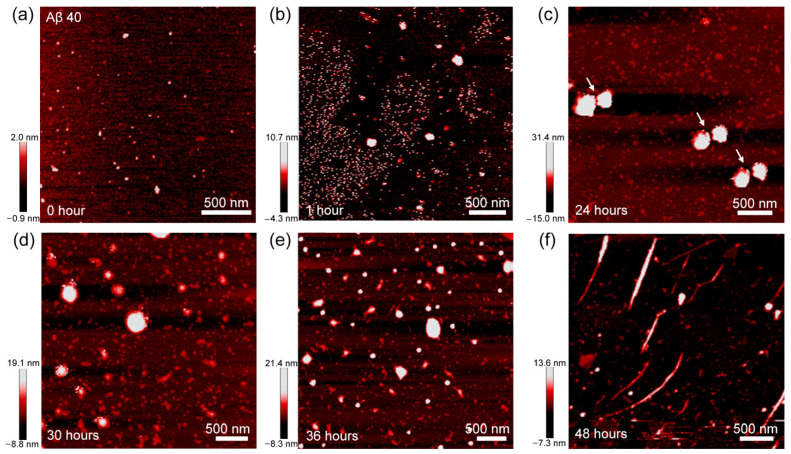
AFM images of Aβ40 aggregates that are made under the incubation time of (**a**) 0 h, (**b**) 1 h, (**c**) 24 h, (**d**) 30 h, (**e**) 36 h, and (**f**) 48 h, respectively. The white arrows indicate two large particles with similar radius are fusing together.

Abundant spherical aggregates are clearly observed for the incubation time of ~0.5 h. Notably, small spherical are found attached to the edges of big aggregates, as indicated by the white arrows in Figure 2b. It should be noted that incubating the sample for 1 h results in the coexistence of few filament-like structures and spherical aggregates (Figure 2c). In fact, a magnified view image (inset of Figure 2c) of these filament structures shows that they are composed by the fusion between spherical aggregates (white arrows in Figure 2c). The attachment between spherical aggregates also can be observed in the sample containing 1 μM Aβ42 (Figure S1b). These data suggest that attachment between sphere-like Aβ42 oligomers possibly is a dominant mechanism guiding the fibrillation process. We also considered the AFM images of Aβ40 aggregates at each fibrillation phase in order to compare with Aβ42 fibrillation process. Since the pre-transition phase of Aβ40 aggregation continued for over 15 h, we collected the AFM image for Aβ 40 at incubation time of 0, 1, 24, 30, 36, and 48 h, respectively (Figure 3a–f).

The big spherical aggregates are observed for 1 h incubation, while they disappeared after 36 h incubation. Surprisingly, we also found similar spherical aggregates and attachments between those aggregates (white arrows in Figure 3c). It is interesting to note that Aβ40 fibrillation induces a heterogeneous distribution of small and large spherical aggregates, rather than forming homogenous one (Figure 3d and 3e). It is energetically favorable for large aggregates to grow faster than the smaller ones [23]. In fact, AFM image for 36 h incubation shows that nearly all large aggregates are fully transformed into short, straight fibrils (Figure 3f). This phenomenon is also found for an Aβ40 sample with a concentration of 1 μM (Figure S1c). Our results propose that fusion between the spherical aggregates is the underlying mechanism of Aβ42 and Aβ40 fibrillation processes.

**Figure 4:**
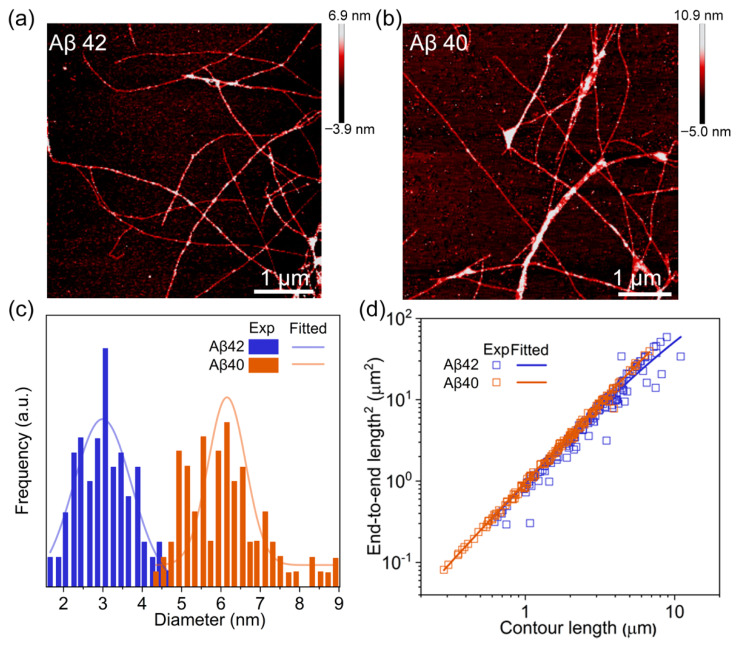
AFM-based microstructure analysis of the formed fibrils. (**a**) AFM image of Aβ42 fibrils obtained at the post-transition stage, showing that Aβ42 monomers are completely transformed into fibrils. (**b**) AFM image of Aβ40 fibrils captured at the post-transition stage. (**c**) The diameter distributions of Aβ42 and Aβ40 fibrils. The blue bar and line denote the experimentally measured data and Gaussian fitting, respectively, for the diameter of Aβ42 fibrils, while the brown bar and line represent the experimental data and Gaussian fitting, respectively, for the diameter of Aβ40 fibrils. (**d**) The relationship between contour length and end-to-end length for Aβ42 and Aβ40 fibrils, respectively, indicated as blue and brown color.

To validate a proposed mechanism of fibrillation, that is, all the spherical aggregates can be converted to fibrils, we consider AFM images of the sample taken from the post-transition stage. The typical AFM images for Aβ42 and Aβ40 aggregations are provided in Figure 4a and b, respectively, unambiguously showing that Aβ monomers are completely converted into fibrils. Here, we note that Aβ42 fibrils exhibit a distinct morphology in comparison with Aβ40 fibrils. Specifically, Aβ42 fibrils are thinner, and occasionally branching at several points along their length (Figure 4a), while Aβ40 fibrils are relatively thicker and they are likely to be bundled (Figure 4b). These two types of fibrils possess the twisted structure, as there are the periodic increases of height for both Aβ42 and Aβ40 fibrils, indicative of twisted structure. In addition, as shown in Figure 4a and b, we can observe more bent configurations for Aβ42 fibrils in comparison with Aβ40 fibrils, which implies that Aβ42 fibrils are more likely to be flexible (see the persistent lengths of these fibrils as discussed in below).

**Figure 5:**
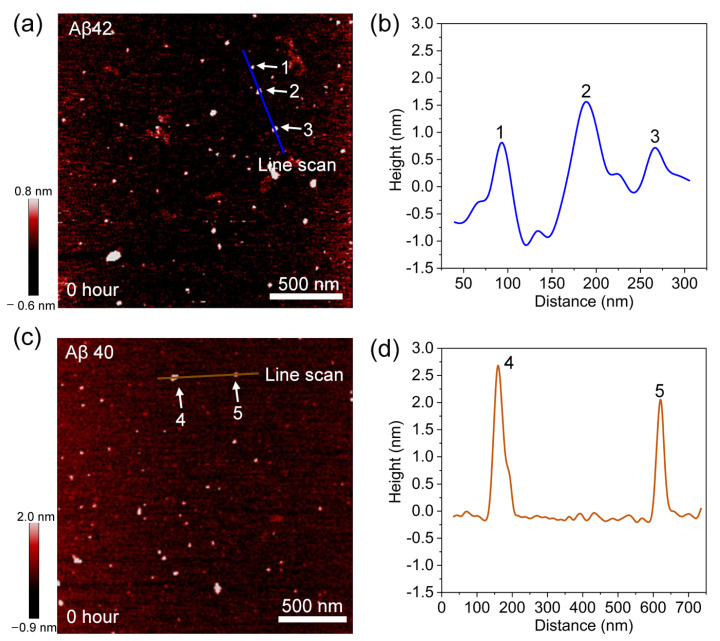
AFM images of oligomers and their height profile. (**a**) AFM image of Aβ42 oligomers. (**b**) Height profile taken along the blue line in (**a**), clearly revealing the height of three different particles. (**c**) AFM image of Aβ40 oligomers. (**d**) Height profile corresponding to the brown line in (**c**).

To quantitatively compare the morphological difference, we investigated the distribution of diameter for more than a hundred Aβ42 and Aβ40 fibrils (Figure 4c). Gaussian fit to the histogram suggests the average diameter of the Aβ42 fibrils to be ~3 nm, markedly narrower than that (i.e., ~6 nm) for Aβ40 fibrils. Additionally, we also measured their persistence lengths based on a worm-like chain model, which provides a relation between the measured contour length (*L*) and the end-to-end distance (*R*). The persistent length is related to the fibril’s bending stiffness, directly revealing the origin of morphology difference between Aβ42 and Aβ40 fibrils. The persistent length of Aβ42 and Aβ40 fibrils is given by 2.08 ± 0.14 and 6.18 ± 0.34 µm, respectively (Figure 4d). The shorter persistence length of Aβ42 fibrils indicates their low stiffness, in good agreement with their observed bent configuration. Indeed, the stiffness difference between Aβ42 and Aβ40 fibrils may be originated from the different Aβ42 and Aβ40 spherical aggregates, which meditate the fibrillation process. We measured the heights of spherical aggregates formed at the early stage of pre-transition phase. Figure 5 shows that the height of three Aβ42 spherical aggregates obtained based on 0 h incubation is equal to ~0.75 or ~1.50 nm. Since the single layered Aβ42 protein has a thickness of 0.75 nm, those spherical aggregates may be made of single- or double-layered Aβ42 proteins. By contrast, the height of Aβ40 aggregates acquired based on 0 h incubation is measured as 2 to 3 nm (Figure 5c and d), suggesting they possibly are small oligomers such as dimers and tetramers [24]. In addition, we measured the diameters of Aβ42 and Aβ40 aggregates obtained from 0 h incubation (Figure S2). The diameter of highly populated Aβ42 oligomers acquired from 0 h incubation is measured as ~10 nm, while the diameter of highly populated Aβ40 oligomers formed based on 0 h incubation is estimated as ~20 nm. The height and diameter measurements suggest the larger Aβ40 oligomers that result in the formation of thicker Aβ40 fibril with its high stiffness when compared with Aβ42 fibrils.

## 4. Conclusions

We present here the direct observation of aggregation pathways of Aβ40 and Aβ42 and their related structures, which remain elusive because of their fast aggregation rate. We slowed down the fibrillation process and directly observed the morphology of Aβ aggregates at each stage of aggregation, clearly capturing the structural features and structural evolution during the process. The monomers are self-assembled to form spherical aggregates at the initial stage with radius depending on the type of Aβ. The Aβ42 aggregation resulted in the formation of small and uniform spherical aggregates, while Aβ40 aggregation induced larger and inhomogeneous spherical aggregates. These spherical aggregates fuse together to form fibrils as incubation time increases, and their size distribution significantly guides the following conversion process and the morphology of final fibrils. The small precipitates give rise to multiple nucleation sites for Aβ42 fibrillation, highly accelerating the fibrillation process. The resultant fibrils have small diameter and are highly flexible. However, Aβ40 spends much longer time to finally convert into thick and stiff fibrils because it forms large aggregates at the initial stage. The contrasting morphology of fibrils and the fibrillation time provide insight into the underlying mechanism of fibrillation process at each stage of aggregation, which may suggest better and more comprehensive picture of molecular mechanism for the onset of neurodegenerative diseases such as AD.

## Data Availability

The data presented in this study are available upon request from the corresponding authors.

## Supplementary Materials

The following are available online at https://www.mdpi.com/2218-273X/11/2/198/s1, Figure S1: ThT spectrum for Aβ40 and Aβ42 aggregation (at a concentration of 1 μM) and their related structures at early stage of aggregation, Figure S2: Diameter distributions for Aβ40 and Aβ42 oligomers.
