# Supplementary material for: Different Aggregation Pathways and Structures for Aβ40 and Aβ42 Peptides
Source: Biomolecules. 2021 Jan 30;11(2):198. doi: 10.3390/biom11020198 (PMC7912290; doi:10.3390/biom11020198)
Supplement: Supplementary file 1 [file biomolecules-11-00198-s001.pdf]

biomolecules

Supplementary materials

Different Aggregation Pathways and Structures for Aβ40 and Aβ42 Peptides

Li Wang[1], Kilho Eom[1,*] and Taeyun Kwon[2,*]

[1]Biomechanics Laboratory, College of Sport Science, Sungkyunkwan University (SKKU), Suwon 16419 and Republic of Korea
[2]SKKU Advanced Institute of Nano Technology (SAINT), Sungkyunkwan University (SKKU), Suwon 16419, Republic of Korea
* Correspondence: should be addressed to K.E. (E-mail: kilhoeom@skku.edu), or T.K. (E-mail: taeyunkwon@skku.edu)

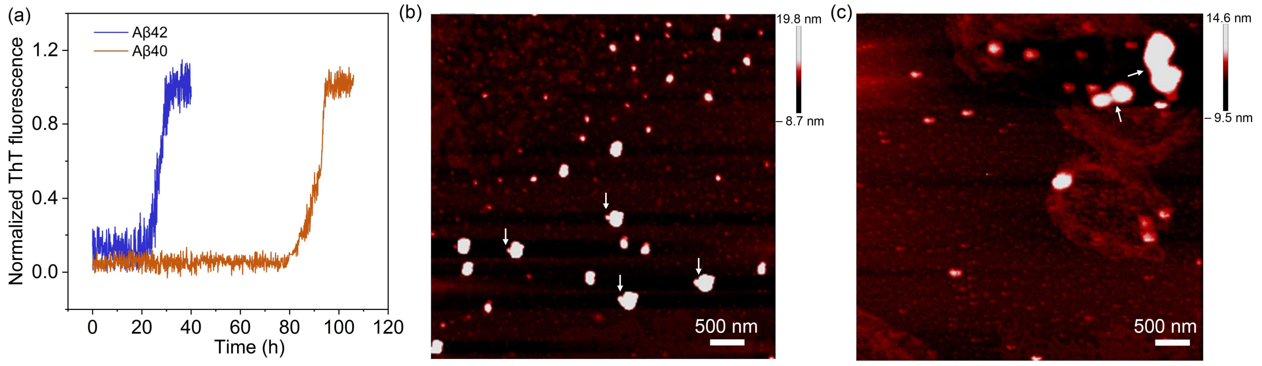

Figure S1. (A) ThT-fluorescence spectrum as a function of incubation time for solution containing 1 μM Aβ42 monomers or 1 μM Aβ40 monomers. **(B)** AFM image taken with a sample containing 1 μM Aβ42. **(C)** AFM image taken with a sample containing 1 μM Aβ40. The white arrows indicate the attachment between the particles.

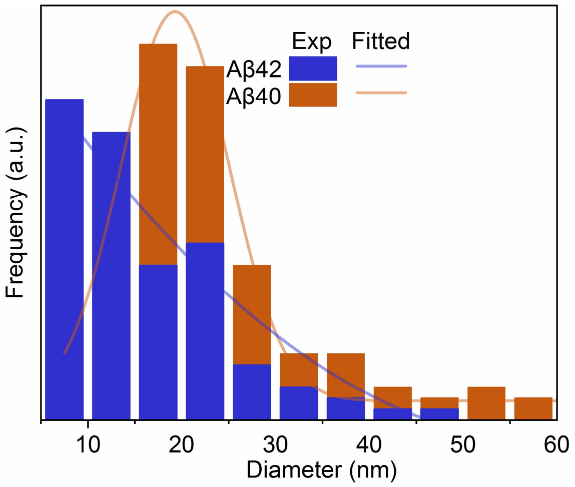

Figure S2. The diameter distribution of oligomers. The blue bar and line represent the experimental data and Gaussian fitting for the size of Aβ42 oligomers obtained based on an incubation of 0 h, while brown bar and line indicate experimental data and Gaussian fitting for the size of Aβ40 oligomers acquired under an incubation of 0 h.
